# Can face masks offer protection from airborne sneeze and cough droplets in close-up, face-to-face human interactions?—A quantitative study

**DOI:** 10.1063/5.0035072

**Published:** 2020-12-01

**Authors:** Javed Akhtar, Abner Luna Garcia, Leonardo Saenz, Sarada Kuravi, Fangjun Shu, Krishna Kota

**Affiliations:** Consortium for Particulate Suspensions, Department of Mechanical and Aerospace Engineering, New Mexico State University, Las Cruces, New Mexico 88003-8001, USA

## Abstract

Day-to-day observations reveal numerous medical and social situations where maintaining physical distancing is either not feasible or not practiced during the time of a viral pandemic, such as, the coronavirus disease 2019 (COVID-19). During these close-up, face-to-face interactions, a common belief is that a susceptible person wearing a face mask is safe, at least to a large extent, from foreign airborne sneeze and cough droplets. This study, for the first time, quantitatively verifies this notion. Droplet flow visualization experiments of a simulated face-to-face interaction with a mask in place were conducted using the particle image velocimetry setup. Five masks were tested in a snug-fit configuration (i.e., with no leakage around the edges): N-95, surgical, cloth PM 2.5, cloth, and wetted cloth PM 2.5. Except for the N-95 mask, the findings showed leakage of airborne droplets through all the face masks in both the configurations of (1) a susceptible person wearing a mask for protection and (2) a virus carrier wearing a mask to prevent the spreading of the virus. When the leakage percentages of these airborne droplets were expressed in terms of the number of virus particles, it was found that masks would not offer complete protection to a susceptible person from a viral infection in close (e.g., <6 ft) face-to-face or frontal human interactions. Therefore, consideration must be given to minimize or avoid such interactions, if possible. This study lends quantitative support to the social distancing and mask-wearing guidelines proposed by the medical research community.

Recent research on face masks shows that they help control the spreading of the respiratory droplets when the wearer sneezes or coughs into the mask.^1–10^ The Centers for Disease Control and Prevention (CDC) and the World Health Organization (WHO) have also stressed on the importance of wearing a mask by both symptomatic and asymptomatic carriers to contain the spreading of viruses, such as, the coronavirus disease 2019 (COVID-19).^11,12^

It can be observed that most of the research conducted so far on face masks has been either qualitative or on respiratory droplets that are >5 *µ*m in size or importantly, from the perspective of a virus carrier wearing a mask to contain the spreading of the virus.^1–10^ From the perspective of a susceptible person wearing a mask for protection, it is only either intuitively believed or qualitatively known that face masks would offer blockage from extraneous matter. At this time, there is no quantitative assessment of the effectiveness of face masks from the perspective of a susceptible person wearing a mask for protection from the airborne sneeze and cough droplets or droplet nuclei (that are <5 *µ*m in diameter).

While it is known that a sneeze or a cough can have both large droplets (>5 *µ*m–10 *µ*m) and droplet nuclei, the droplet nuclei can linger in the air much longer.^13–17^ These <5 *μ*m-sized droplets or nuclei can escape the pores of face masks more easily than the large droplets. It was found that particles that are <200 nm in size are often captured by filters (such as masks, for example) due to the Brownian motion (e.g., Ref. 18). Therefore, the droplet nuclei, whose size falls in this intermediate range between the sub-micron virus particles and the large droplets, pose a challenge to the mask material’s filtration ability.

In addition to wearing face masks (and frequent soap-assisted washing of hands), social distancing is also suggested by the CDC and WHO for controlling the spreading of respiratory droplets and nuclei from a virus carrier. However, day-to-day observations reveal numerous medical and social situations where maintaining physical distancing is either not feasible or not practiced, e.g., either in closed spaces, such as hospitals, homes, gymnasiums, public transportation, and schools, or physically close interactions in indoor and outdoor spaces, such as in crowded gatherings at organized events and political campaigns. People will eventually start to socialize and travel to combat depression. Restaurants will allow indoor dining, and airliners will eventually allow middle-seat occupancy; all of these will increase the number of close-up human interaction scenarios even more (e.g., Ref. 19).

In many such close face-to-face or frontal interaction scenarios, a common belief appears to be widely pervading that a susceptible person wearing a face mask is safe, at least to a large extent, from foreign sneeze and cough droplets. This study verifies this notion using particle image velocimetry (PIV)-based counting of particles. Accordingly, this study quantitatively answers the following questions:

1. How much protection will a face mask offer to the wearer—such as a frontline worker, a caregiver, an airline passenger, or a diner—from foreign airborne droplets in the close vicinity of a sneeze or a cough?
2. How effective is a face mask in reducing the spreading of droplets (source control) when the wearer sneezes or coughs into it?
3. Can a facemask be made more effective by wetting it with water?

An experimental setup was built to answer these questions, and the particle image velocimetry (PIV) setup was employed to visualize and count the number of simulated droplets (Fig. 1). The setup was designed to simulate direct frontal exposure, which would put the mask materials’ ability to a stringent test.

**FIG. 1.**
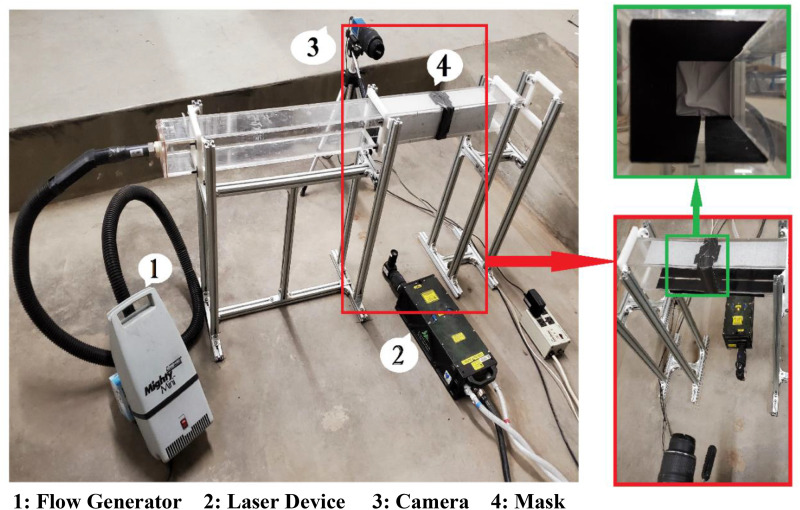
PIV-based flow visualization setup was used for counting the number of simulated sneeze or cough droplets that escape through a snugly fit mask.

Two 0.25 in.-thick transparent acrylic glass square tubes, 34 in. and 12 in. long, of 3 in. × 3 in. internal cross section were joined with a mask snugly fit between them. Air-tight sealing of the junction with the mask was achieved using duct tape. An aluminum stand was built to fix the tubes above the ground and allow space to position a laser under the tubes for visualization. The camera lens was placed in parallel to the laser sheet plane such that the masks are located at the center of the captured images. The PIV system is composed of a Nd:YAG laser with a wavelength of 532 nm, a charge-coupled device (CCD) camera, an external trigger, and DaVis 8.2 software for image processing. A temporal resolution of 3 Hz was used for the image acquisition, and 100 pairs of images were secured for each mask experiment for capturing (and counting) the particles on both sides of the mask.

To avoid saturation of brightness and suppress reflection and boundary glare, the square tubes’ top and back walls (from the perspective of the camera) were completely blackened. The bottom wall was also blackened except for a thin slot for allowing the laser sheet to pass. A flow generator was attached to one end of the longer tube for generating airflow with the simulated droplets of water. A centerline velocity of 3.2 m/s was used in all the experiments. This value, as an average, captures most of the typical sneeze and cough scenarios in close (<6 ft) frontal exposure situations.^20,21^

Given the scope of this study, the size of the particles is an important parameter. It was observed in extensive independent analyses of various human exhalations that the size of the droplets—that are more prone to be airborne and thereby travel with moving air to a person nearby—is on the order of 1 *µ*m.^22,23^ A Laskin nozzle particle generator, which is widely used in flow visualization experiments, was used for creating the 1 *μ*m-sized droplets in all the experiments.

For ease of interpretation, the post-processed data are provided in combination with the flow visualization images in Fig. 2. For the purpose of presentation in this article, all the images were uniformly brightened. In the figure, Escape Percentage (EP) denotes the percentage of droplets from a sneeze or cough that escaped or traveled through the mask material. On the contrary, a mask’s effectiveness in preventing the droplets from escaping or traveling through it can be obtained as 100 − EP.

**FIG. 2.**
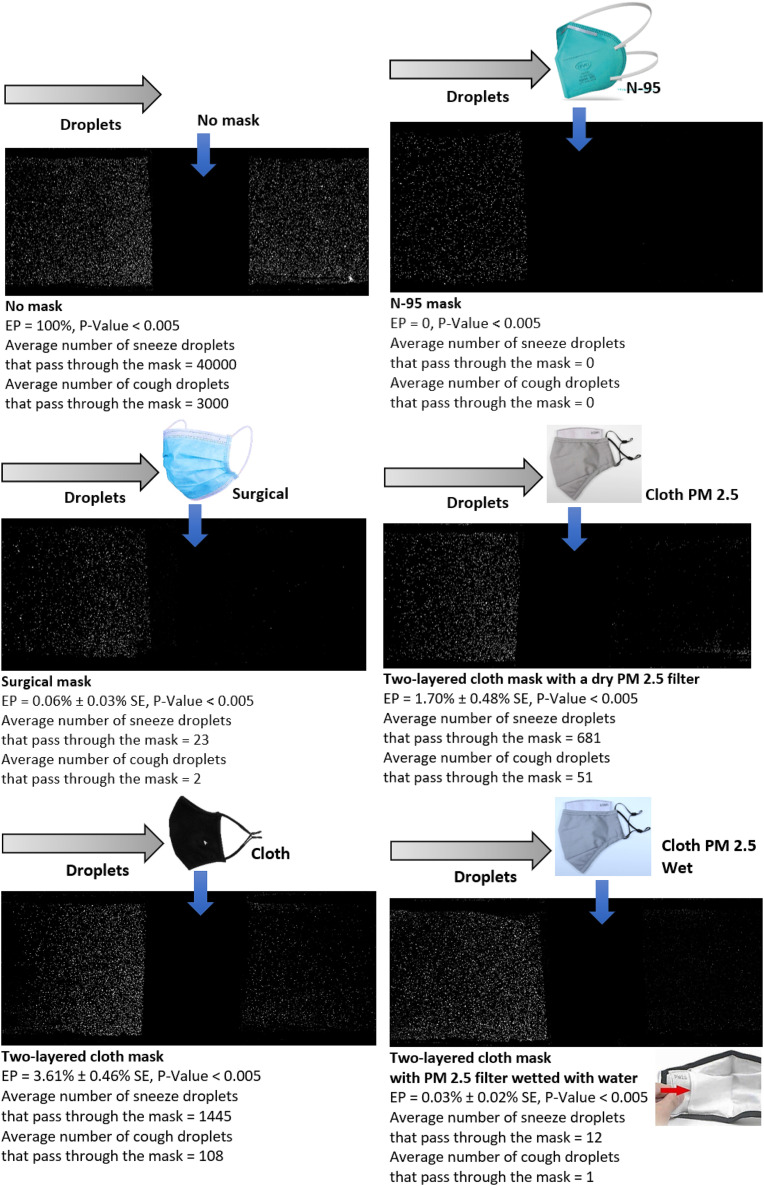
Results of the study. EP denotes the Escape Percentage, i.e., the percentage of droplets from a sneeze or cough that could escape or travel through a snugly fit mask. A mask’s effectiveness in preventing the airborne droplets from escaping or traveling through it can be obtained as 100 − EP. A typical sneeze and a cough are assumed to contain 40 000 and 3000 droplets, respectively.^20–23^

For providing the readers with an understanding of the practical impact of EP, the results are also presented in terms of the average number of foreign sneeze and cough droplets that can escape a mask in the considered close (<6 ft) face-to-face interaction scenario. These numbers are based on multiple studies that stated that a typical sneeze and a cough could contain 40 000 and 3000 droplets, respectively.^20–23^

Studies have shown that the viral infection threshold, e.g., for COVID-19, for a susceptible person is 1000 virus particles, inhaled either at once or in batches.^24,25^ Therefore, Fig. 3 shows the tested masks’ filtering ability in keeping the virus particle concentration below this threshold. The average mask EP was used as the basis. It is published in the literature that a single sneeze can contain anywhere from a few tens of millions to 200 × 10^6^ virus particles depending on the virus concentration (or load) of the carrier.^22,24^ To cover a wide range of virus concentration scenarios—either due to the load or physical distance—the mask EP values were shown assuming 10 000 to 200 × 10^6^ virus particles per one cough or sneeze.

**FIG. 3.**
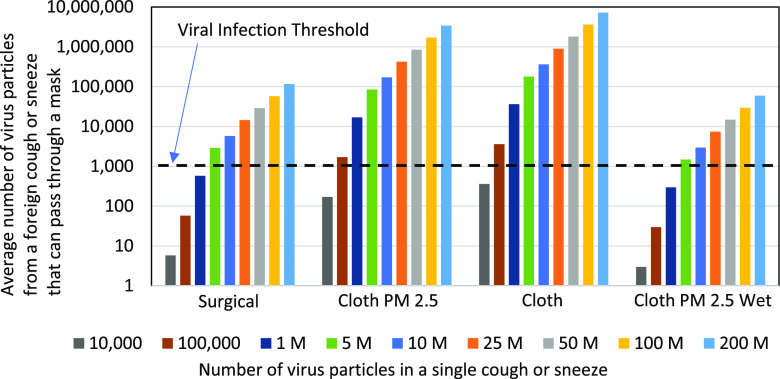
The graph shows the average number of virus particles that can pass through the mask of a susceptible person when exposed to a single cough or sneeze from a virus carrier in a close (<6 ft) face-to-face interaction. Studies have shown that the infection threshold for a susceptible person to catch a virus, such as COVID-19, is 1000 virus particles, inhaled either at once or in batches.^24,25^ Since the N-95 mask has statistically zero particles escaping through it in the “protection” configuration, it was excluded from this figure. However, in the “source control” configuration, the cutoff for the N-95 mask could be as low as 100 000 virus particles based on its average EP value.

Finally, another set of experiments was also conducted to measure the mask effectiveness if a virus carrier is wearing a mask (assuming a snug fit) and sneezes or coughs into it. The mask direction was reversed to simulate these experiments to measure the difference, if any, in the EP. The EP values are as follows: N-95: 0.98% ± 0.69% SE, Surgical: 0.19% ± 0.06% SE, Cloth PM 2.5: 0.19% ± 0.06% SE, Cloth: 2.7% ± 0.2% SE, and Cloth PM 2.5 Wet: 0.07% ± 0.06% SE. Since the mask in this scenario in real life will be closer to the ejection or exhalation source (mouth or nose), the velocity could be 3–4 times larger than 3.2 m/s.^20–23^ Furthermore, due to this increased velocity, this setting could have a large edge leakage effect. It implies that it is possible that a larger number of airborne droplets, than the EP values provided above, could escape the masks and travel toward a susceptible person. The EP value for the N-95 mask is an outlier or could mean that N-95 is more effective in blocking airborne matter coming from the outside than that coming from the inside of it (e.g., as worn by fire safety personnel). The differences in the EP values for this “source control” scenario compared to those measured in the “protection” scenario could be attributed, among other factors, to a difference in the arrangement, material, and the stretching of the clothing layers that could generate a different porous path (e.g., tortuosity) for the flow when the mask is reversed.

From the obtained data and the data deduced using published work on the viral and flow characteristics of sneezes and coughs (as shown in Figs. 2 and 3), the conclusions and recommendations from this study are summarized below. As a reminder, it is noted that these conclusions are more applicable for airborne droplets (nuclei) in close (<6 ft) face-to-face or frontal interaction situations.

1. Without a face mask, it is almost certain that many foreign droplets will transfer to the susceptible person. Wearing a mask will offer substantial, but not complete, protection to a susceptible person by decreasing the number of foreign airborne sneeze and cough droplets that would otherwise enter the person without the mask.
2. Consideration must be given to minimize or avoid close face-to-face or frontal human interactions, if possible. If the relevant social distancing guidelines are compromised, the study shows that foreign airborne sneeze and cough droplets could pass through all the masks tested (except for the N-95 mask) even when assuming a 100% snug fit.
3. Studies show that a single sneeze can usually contain 10–200 × 10^6^ virus particles depending on the carrier’s virus load. In close-up, face-to-face interactions, factors that could dilute the virus concentration [such as diffusion of the droplets or some of the large (>5 *µ*m–10 *µ*m) droplets falling to the ground] will be less important. Even when the concentration of the virus particles in a single sneeze or cough is less than 10 × 10^6^, the results show that none of the masks tested would be able to offer protection to the susceptible person. Depending on the mask’s type, they appear to be effective only when the susceptible person is exposed to virus concentrations of less than 5 × 10^6^ per sneeze or cough for surgical and cloth PM 2.5 wet masks. This cutoff is as low as less than 100 000 for the dry cloth masks. If the edge leakage factor of the masks is taken into account, these cutoffs could be even lower. Such low concentrations could be possible only when there is sufficient and rapid dispersion of a sneeze or cough in the surrounding environment, or if the virus load of the carrier is very low. In a study that modeled airborne droplet transmission, it was found that saliva droplets (with a decrease in the droplet concentration and size) could travel as far as 19 ft–20 ft in the wind direction for wind speeds varying from 1.1 m/s to 4.1 m/s.^26^ In both the indoor and the outdoor (especially windy) environments, high relative humidity coupled with the airflow direction and speed could carry the virus particles either away from a mask or bring them toward the mask to cause frontal exposure.^26,27^
4. The cloth PM 2.5 mask wetted with water has exhibited a better performance in blocking the airborne droplets than all the other masks tested (except for the N-95 mask), including the surgical mask. If a mask is wetted, the material’s fibers will swell, reducing the pore size available for the droplets to penetrate through the mask. The wet mask might have to be disposed or occasionally washed to replenish with freshwater to address the virus saturation and water evaporation concerns. The wet mask approach could prove useful in medical environments where it could be disposed of after each interaction with the virus carrier.
5. The experiments that simulated the scenario of a virus carrier wearing a mask show that many small sneeze or cough droplets can still escape a mask even when the mask is snugly fit. Therefore, both the virus carrier and the susceptible person could consider wearing a mask to reduce the transmission and spread of the virus.
6. If close-up contact cannot be avoided, such as in frontline worker activities, some ideas could be: using wet masks or turning the face away from the sneeze or cough or both. Such additional measures could at least lower the risk to some extent by avoiding a direct frontal exposure to the droplets and possibly providing a longer time for the diffusion/dilution of the airborne droplets to occur. Some medical experts and prior research also suggest the second option (e.g., Refs. 28 and 29). Furthermore, depending on breathing comfort, wearing multiple layers of masks could offer increased protection (e.g., Ref. 1).
7. This study, which can be treated as precautionary, provides quantitative support to the guidelines proposed by the medical research community that wearing a mask and avoiding close face-to-face or frontal interactions as much as possible will help in preventing the transmission and spreading of virus particles, such as COVID-19, through sneezes and coughs.

One study projects more than 500 000 COVID-19 deaths in the United States (US) by early next year, and close-up interactions and the inconsistent use of masks are cited as some of the possible reasons.^30,31^ The US and Europe have recorded their days of the highest number of COVID-19 cases (83 757 and 200 000, respectively) in October 2020.^30,31^ In November 2020, the number of COVID-19 cases in a single day have crossed 160 000 in the US.^32^ This study reveals that masks are not entirely fool-proof in blocking the airborne droplets even if they are snugly fit. Hence, it can be assumed that the high infection numbers could be, among other factors, such as increased testing and the weather, a result of people starting to socialize through close-up, face-to-face interactions even if they are wearing a mask.

## AUTHORS’ CONTRIBUTIONS

J.A., A.L.G., and L.S. have worked on building the experimental setup, statistical post-processing of the data, and setting up the micro-PIV experiment, respectively. All three of them have equally contributed to conducting the experiments and obtaining the data. S.K., F.S., and K.K. have participated in the analysis of the data. S.K. and K.K. have contributed to writing the paper, and F.S. has reviewed it. F.S. and K.K. have conceived the experiments. K.K. has initiated the work.

## Data Availability

The raw data obtained from this study are available with the authors and will be provided upon request. The post-processed data are available in the manuscript.
